# The complexities of MCS – How far is too far?

**DOI:** 10.1051/ject/2025012

**Published:** 2025-06-16

**Authors:** Salman Pervaiz Butt, Drisya Paul, Umer Darr, Gopal Bhatnagar

**Affiliations:** 1 Perfusion Services, Heart Vascular and Thoracic Institute, Cleveland Clinic Abu Dhabi PO BOX 112412 Abu Dhabi United Arab Emirates; 2 Cardiac Surgery, Heart Vascular and Thoracic Institute, Cleveland Clinic Abu Dhabi PO BOX 112412 Abu Dhabi United Arab Emirates

Dear Editor,

Mechanical circulatory support (MCS) devices, including right ventricular assist devices (RVADs), left ventricular assist devices (LVADs), Impella, extracorporeal membrane oxygenation (ECMO), and intra-aortic balloon pumps (IABPs), have transformed the management of advanced heart failure, cardiogenic shock, and severe respiratory failure. These technologies offer critical support and have saved countless lives. However, as the use of these devices, often in combination, becomes more common, we face increasing ethical, clinical, and societal challenges, particularly due to the lack of long-term outcome data for patients supported with multiple devices. Thoughtful consideration is essential as we continue to expand their use in complex clinical scenarios.

The central question we face is: How far is too far? This becomes particularly significant in high-income countries, where resource limitations are less of a concern. In such settings, caregivers may be inclined to use every available technological intervention, often under the assumption that the more extreme the intervention, the better the outcome [1]. This can lead to a chain of interventions LVAD followed by RVAD, then Impella or IABP without fully considering the associated morbidity, mortality rates, and overall quality of life for the patient. Literature on the use of multiple MCS devices in succession suggests that while these devices may provide short-term support, their combined use can significantly increase complications, including infections, organ dysfunction, and long-term recovery challenges. Timely implementation of these devices also decides the patient’s recovery. Studies have also reported that the sequential use of multiple MCS devices can lead to a high incidence of adverse outcomes, underscoring the need for caution and careful patient selection. A survival gap persists in destination therapy compared to bridge patients. RVAD need and delay impact survival dramatically [2]. Furthermore, while these interventions may extend life, they raise important questions about the balance between prolonging life and sustaining meaningful quality of life.

The combination of IABP and Impella devices in patients undergoing percutaneous coronary intervention (PCI) has been linked to higher rates of bleeding complications and in-hospital mortality compared to using either device alone [3]. This highlights the potential risks of combining multiple MCS devices, which may overwhelm the body’s ability to cope with the associated complications, ultimately leading to worse clinical outcomes. It is evident for patients, particularly those with limited overall prognosis, that the use of multiple devices can lead to a prolonged and often burdensome hospital course, rather than recovery.

Neurological complications, including acute brain injuries, stroke is common in both short-term and durable MCS applications particularly in the early post-heart transplantation period. These risks significantly impact the quality of life and long-term survival, making it essential for clinicians to carefully consider the appropriateness of MCS devices, especially when combined [4].

The ethical considerations surrounding the weaning or deactivation of life-sustaining devices are complex. On one hand, medical technology can extend life, on the other, it can prolong suffering, particularly when the quality of life is severely compromised. The decision to discontinue or withhold treatment is not only a medical challenge but also a moral one, requiring a delicate balance between prolonging life and respecting patient dignity [5]. In such cases, shared decision-making becomes crucial where doctors, caregivers, and family members must collaborate to determine what the patient would have wanted, considering both medical prognosis and quality of life.

As the field of MCS evolves with advancements like minimally invasive devices, fully implantable systems, and bioengineered solutions, we must question whether rapid progress is outpacing our understanding of long-term consequences, as constant modifications to circuits, facilitated by easy access to technology, may increase mortality and stroke complications [6].

While innovation is vital, it must be guided by thorough clinical evaluation, ethical consideration, and a strong focus on patient-centered care. The rapid advancement and widespread use of extracorporeal technologies such as ECMO, often applied in situations where long-term outcomes remain uncertain and have introduced complex ethical challenges [7]. These devices, when used beyond clearly defined indications, risk shifting from life-saving interventions to uncertain destination therapies. As clinicians, researchers, and policymakers, it is our responsibility to maintain an ongoing dialogue that ensures the use of mechanical circulatory support is not driven solely by technological capability, but by thoughtful, ethical, and patient-focused decision-making.
